# Outcomes of suture-assisted gonioscopy-assisted transluminal trabeculotomy in a cohort of South African patients with primary open-angle glaucoma

**DOI:** 10.4102/jcmsa.v4i1.418

**Published:** 2026-09-24

**Authors:** Walid Huwaidi, Mansour Wasl, Christopher Tinley, David Steven

**Affiliations:** 1 Division of Ophthalmology, Faculty of Medicine and Health Sciences, University of Cape Town, Cape Town, South Africa

**Keywords:** gonioscopy-assisted transluminal trabeculotomy, primary open-angle glaucoma, minimally invasive glaucoma surgery, intraocular pressure, phacoemulsification, hyphema, South African patients, South Africa

## Abstract

**Background:**

Gonioscopy-assisted transluminal trabeculotomy (GATT) is a minimally invasive glaucoma surgery that enhances trabecular outflow by circumferentially incising Schlemm’s canal. Internationally, studies have reported favourable outcomes, but evidence from South African patients remains limited. This study evaluated the effectiveness and safety of GATT in South African patients with primary open-angle glaucoma (POAG).

**Methods:**

A retrospective cohort study was conducted at Eerste River Hospital. Patients with POAG who underwent GATT between August 2020 and November 2022 were followed for 24 months. They were divided into two groups: combined phacoemulsification and GATT (Group I) and standalone GATT (Group II). Demographic characteristics, intraocular pressure (IOP), glaucoma medication use, and postoperative complications were collected.

**Results:**

Fifty-two patients (*n* = 52) were included. Mean preoperative IOP for the entire cohort was 23.4 ± 6.4 mmHg and decreased significantly to 13.8 ± 4.2 mmHg at 24 months (*p* = 0.006). Mean glaucoma medication use also decreased, from 3.5 ± 0.6 preoperatively to 2.5 ± 1.1 at 24 months (*p* < 0.001). The cumulative success rate at 24 months was 94.23%. Complications were mostly transient, with hyphema being the most frequent. No serious adverse events were reported.

**Conclusion:**

Gonioscopy-assisted transluminal trabeculotomy, performed alone or combined with phacoemulsification, was an effective and safe option for lowering IOP in South African patients with POAG. The sustained IOP reduction, decreased medication burden and favourable safety profile support its use, particularly in resource-limited settings.

**Contribution:**

This study provides region-specific evidence on the outcomes and safety of GATT in a South African POAG cohort.

## Introduction

Glaucoma, a chronic and progressive optic neuropathy, is a leading global cause of irreversible blindness. It is characterised by distinct damage to the optic nerve and corresponding visual field loss, most frequently associated with elevated intraocular pressure (IOP).^1^ Projections, published in 2006, estimated that 60.5 million people would be living with glaucoma in 2010, increasing to nearly 79.6m by 2020.^2^ More recent analysis extended these projections, suggesting that the global burden of glaucoma may rise to 111.8m individuals by 2040.^3^ Among the various forms of the disease, primary open-angle glaucoma (POAG) has been consistently identified as the most prevalent subtype worldwide.^4^ In South Africa, epidemiological studies demonstrate that POAG represents the dominant form of glaucoma, carrying a substantial risk of irreversible visual impairment if not detected and treated early.^5^

The underlying pathophysiology of POAG is characterised by increased resistance to aqueous humour outflow, primarily at the trabecular meshwork and Schlemm’s canal, leading to elevated IOP and progressive optic nerve damage, with corresponding visual field loss.^1,6^ Critically, the reduction of IOP remains the primary evidence-based strategy proven to effectively slow the progression of this sight-threatening disease, achievable through pharmacological agents, laser therapy or surgical intervention.^1,7^ While topical hypotensive medications are typically the initial treatment modality, their long-term efficacy is frequently limited by challenges such as poor patient adherence, significant cost implications and undesirable side effects.^7^ Similarly, laser trabeculoplasty, though offering temporary IOP reduction, often demonstrates a decline in effectiveness over time.^7^

For patients requiring more definitive surgical intervention, conventional procedures like trabeculectomy and glaucoma drainage devices (GDDs) have served as the historical mainstays for decades.^8^ Despite their proven effectiveness in lowering IOP, these traditional surgeries are unfortunately associated with considerable risks, including the potential for hypotony, various bleb-related complications and long-term surgical failure, largely due to excessive fibrosis.^7,8^ These limitations have stimulated the evolution and increased adoption of minimally invasive glaucoma surgery (MIGS) techniques, which present safer alternatives, characterised by fewer complications, reduced invasiveness and typically faster patient recovery.^7,9^

Among the innovative MIGS techniques, gonioscopy-assisted transluminal trabeculotomy (GATT) is an ab interno procedure, first described by Grover et al. in 2014.^10^ This technique involves performing a circumferential trabeculotomy through Schlemm’s canal utilising a microcatheter or suture, facilitated by a clear corneal incision under direct gonioscopic visualisation.^10,11^ A key advantage of GATT is its ability to spare the conjunctiva and avoid the formation of external filtering blebs, thereby preserving future surgical options for the patient while significantly reducing the associated risk profile compared with traditional filtration surgeries.^10,11^ The mechanism by which GATT lowers IOP is fundamentally physiological; it improves the natural aqueous humour outflow through the conventional pathway by directly bypassing the diseased trabecular meshwork, which is the primary site of outflow resistance in POAG.^6^ The common and typically transient postoperative finding of micro-hyphema, a small amount of blood in the anterior chamber, is often considered a positive prognostic indicator, thought to reflect the successful restoration of this outflow pathway.^12,13^

Numerous studies across high-income countries have consistently demonstrated that GATT achieves significant reductions in both IOP and the number of glaucoma medications required in patients presenting with mild to advanced POAG.^9,10,14,15^ Its efficacy is comparable to that of conventional surgeries and GDDs in various settings.^9,14,15^

Furthermore, GATT has yielded favourable outcomes in specific patient populations, including those with juvenile open-angle glaucoma,^10,16^ congenital glaucoma^16,17^ and steroid-induced glaucoma.^9,18^ Its adaptability, allowing for successful performance using both microcatheters and a polypropylene suture, also makes it a practical and viable option for resource-limited healthcare environments.^11,14^

The combination of GATT and phacoemulsification for cataract removal has consistently been shown to provide an additive IOP-lowering effect.^19,20,21,22,23^ This combined approach addresses both glaucoma and visually significant cataracts in a single surgical session. While cataract surgery alone can lead to a modest reduction in IOP by deepening the anterior chamber and widening the drainage angle,^19,24^ this effect is often insufficient for managing moderate to severe glaucoma.^24^ Consequently, integrating cataract extraction with MIGS techniques such as GATT offers a comprehensive and efficient surgical solution for suitable patients.^20,21,22,23^

Despite the growing body of evidence supporting GATT in developed countries, there remains a notable lack of data regarding its effectiveness and safety within South African patients. A critical South African prevalence study, older but foundational, highlighted the substantial burden of both POAG and primary angle-closure glaucoma in regions like the Western Cape.^5^ In this specific demographic, the attributes of GATT in terms of its cost-effectiveness, favourable safety profile and conjunctiva-sparing nature position it as a potentially invaluable intervention in the comprehensive management of glaucoma, particularly relevant for resource-constrained settings.

Therefore, this study aims to evaluate the real-world outcomes of GATT in a cohort of South African patients diagnosed with POAG. Additionally, it will compare the efficacy and safety of GATT performed alone versus GATT combined with phacoemulsification, assessing key parameters such as IOP reduction, changes in glaucoma medication reliance and the incidence of postoperative complications over a 2-year follow-up period.

## Research methods and design

This retrospective cohort study included patients with POAG who underwent GATT at Eerste River Hospital, Cape Town, between August 2020 and November 2022.

All surgical procedures were performed by a single surgeon and conducted under sub-Tenon’s anaesthesia. Gonioscopy-assisted transluminal trabeculotomy was performed ab interno using a 5.0 polypropylene suture to achieve a circumferential trabeculotomy. Patients were stratified into two groups: Group I (*n* = 41) underwent GATT combined with phacoemulsification and intraocular lens (IOL) implantation, while Group II (*n* = 11) underwent GATT as a standalone procedure. In Group II, six patients were phakic, and five were pseudophakic at the time of surgery.

All patients were prescribed topical broad-spectrum antibiotics and topical corticosteroids. Antibiotic therapy was discontinued at postoperative week 4, while steroid drops were tapered over 1 to 2 months, with the primary aim of controlling inflammation and minimising the risk of steroid-induced IOP response.

Intraocular pressure was measured using Goldmann applanation tonometry, which remains the gold standard for clinical assessment. Measurements were performed by trained registrars and consultants during routine clinic hours (08:00–16:00). Although central corneal thickness (CCT) was recorded as part of the routine preoperative glaucoma assessment, IOP values were not adjusted for CCT.

### Inclusion criteria

Patients with POAG who underwent GATT between 01 August 2020 and 31 December 2022 were included in the study. All patients had open angles according to the Shaffer classification. The primary indication for GATT surgery was inadequately controlled IOP despite maximal tolerated medical therapy. Cataract surgery was performed concurrently when clinically significant lens opacities were noted on preoperative evaluation.

### Exclusion criteria

Patients were excluded from the study if they had a history of previous glaucoma surgery, incomplete or missing follow-up data, co-existing ocular pathologies or were receiving anticoagulant medication.

Quantitative data, such as age, IOP and the number of glaucoma medications, were expressed as either mean ± standard deviation (s.d.) and range or as frequencies and percentages.

For comparisons of continuous variables between the two independent groups (Group I: GATT combined with phacoemulsification surgery; Group II: GATT alone), independent samples *t*-test was utilised. For categorical variables like sex, chi-square tests were employed to assess differences in proportions between the groups.

Longitudinal changes in IOP and the number of glaucoma medications within each group over the 2-year follow-up period were assessed using repeated measures analysis of variance (ANOVA) or equivalent nonparametric tests, where appropriate. Comparisons of IOP and medication use between Group I and Group II at various post-operative time points were conducted using independent samples *t*-tests.

A *p*-value of less than 0.05 was considered statistically significant for all analyses.

Kaplan–Meier survival analysis was performed to evaluate the cumulative success rate, defined as controlled IOP without the need for further glaucoma surgery.

### Bias

This was a retrospective study; therefore, selection bias and information bias are possible. To minimise selection bias, we included only patients with regular follow-up. Information bias was reduced by using standardised measurements (all procedures were performed by the same surgeon) that were consistent across both groups.

### Ethical considerations

Ethical clearance to conduct this study was obtained from the University of Cape Town Health Sciences Human Research Ethics Committee on 09 November 2023 (No: 812/2023). All information was anonymised and stored on a secure, password-protected device.

## Results

### Baseline demographics

A total of 52 patients with POAG were evaluated in this study: 41 patients in Group I (GATT combined with phacoemulsification) and 11 patients in Group II (GATT alone). The baseline clinical and demographic characteristics of both study arms are summarised in Table 1. The mean age was 62.1 ± 9.4 years (range: 34–77 years) in Group I and 56.5 ± 16.6 years (range: 36–80 years) in Group II (*p* = 0.146). Gender distribution showed 22 males (53.7%) in Group I and eight males (72.7%) in Group II (*p* = 0.256).

**TABLE 1:** Demographic data among study patients.

Characteristic	Group I (*n* = 41)				Group II (*n* = 11)				*p*-value
	Mean ± s.d.	Range	*n*	%	Mean ± s.d.	Range	*n*	%	
Age (years)	62.1 ± 9.4	34–77	-	-	56.5 ± 16.6	36–80	-	-	0.146
**Sex**	-	-	-	-	-	-	-	-	0.256
Male	-	-	22	53.7	-	-	8	72.7	-
Female	-	-	19	46.3	-	-	3	27.3	-

s.d., standard deviation.

### Intraocular pressure and medication burden

Preoperative baseline values for IOP (*p* = 0.517) and the number of glaucoma medications (*p* = 0.353) did not differ between the two groups (Table 2).

**TABLE 2:** Comparison between the two studied groups as regards intraocular pressure and number of glaucoma medications.

Time point	Parameter	Group I (*n*= 41)	Group II (*n* = 11)	*p*-value
Pre-operative	IOP	23.7 ± 6.1	22.3 ± 7.7	0.517
	Treatment	3.5 ± 0.6	3.6 ± 0.5	0.353
1st day	IOP	13.7 ± 4.9	12.4 ± 4.3	0.421
	Treatment	0	0	-
1st week	IOP	16.9 ± 6.3	17.2 ± 11.6	0.911
	Treatment	0.2 ± 0.8	0	0.426
1st month	IOP	16.4 ± 6.2	17.9 ± 6.4	0.499
	Treatment	0.5 ± 0.9	0.4 ± 1.2	0.702
3rd month	IOP	14.6 ± 3.2	15.5 ± 5.6	0.650
	Treatment	0.9 ± 1.2	1.3 ±1.9	0.574
6th month	IOP	14.9 ± 4.6	13.9 ± 4.1	0.572
	Treatment	1.3 ± 1.2	2.4 ± 1.2	0.019
9th month	IOP	14.9 ± 4.1	11.3 ± 5.6	0.215
	Treatment	1.31 ± 1.3	3 ± 1	0.017
1st year	IOP	15.2 ± 3.8	14 ± 3.9	0.620
	Treatment	1.2 ± 1.4	2 ± 1.9	0.259
2nd year	IOP	13.5 ± 3.7	14.5 ± 5.5	0.874
	Treatment	2.4 ± 1.3	2.8 ± 0.7	0.182

IOP, intraocular pressure.

In Group I, the mean preoperative IOP decreased from 23.7 mmHg ± 6.1 mmHg to 13.7 mmHg ± 4.9 mmHg on postoperative day 1. At 1 week, the mean IOP was 16.9 mmHg ± 6.3 mmHg, which progressively stabilised to 15.2 mmHg ± 3.8 mmHg at 1 year and 13.5 mmHg ± 3.7 mmHg at 2 years (*p* = 0.003). The mean number of daily glaucoma medications in Group I dropped from a preoperative baseline of 3.5 ± 0.6 to 0 on day 1, 0.2 ± 0.8 at 1 week, 1.2 ± 1.4 at 1 year and 2.4 ± 1.3 at 2 years (*p* < 0.001).

In Group II, the mean preoperative IOP decreased from 22.3 mmHg ± 7.7 mmHg to 12.4 mmHg ± 4.3 mmHg on day 1, and was 17.2 mmHg ± 11.6 mmHg at 1 week. Over the remaining follow-up intervals, the mean IOP values were 14.0 mmHg ± 3.9 mmHg at 1 year and 14.5 mmHg ± 5.5 mmHg at 2 years (*p* = 0.144). Glaucoma medication requirements in Group II shifted from a preoperative mean of 3.6 ± 0.5 to 0 at 1 week, 2.0 ± 1.9 at 1 year and 2.8 ± 0.7 at 2 years (*p* = 0.090).

Inter-group comparisons demonstrated *p* > 0.05 across all time points up through the 2-year endpoint. Regarding medication burden, Group I required significantly fewer medications than Group II at the 6-month (1.3 ± 1.2 vs. 2.4 ± 1.2; *p* = 0.019) and 9-month follow-ups (1.31 ± 1.3 vs. 3.0 ± 1.0; *p* = 0.017). However, medication burdens between the two groups were equivalent at the 1-year (*p* = 0.259) and 2-year (*p* = 0.182) intervals.

### Postoperative complications

Transient postoperative complications are outlined in Table 3. Microscopic hyphema occurred in 16 eyes (39.0%) in Group I and two eyes (18.2%) in Group II (*p* = 0.30). Macroscopic hyphema was recorded in nine eyes (22.0%) in Group I and five eyes (45.5%) in Group II (*p* = 0.26). All cases of hyphema resolved spontaneously within the early postoperative weeks without surgical intervention. Other complications, including fibrinous uveitis and transient hypotony, occurred in a small proportion of eyes and showed comparable rates between both groups (*p* > 0.05). No cases of endophthalmitis, suprachoroidal haemorrhage or permanent vision-threatening complications were encountered.

**TABLE 3:** Postoperative complications in both groups.

Complication	Group I (*n* = 41)		Group II (*n* = 11)		*p*-value
	*n*	%	*n*	%	
Microscopic hyphema	16	39.0	2	18.2	0.30
Macroscopic hyphema	9	22.0	5	45.5	0.26
Fibrinous uveitis	3	7.3	1	9.1	> 0.99
Transient hypotony	2	4.9	1	9.1	0.51

### Kaplan–Meier survival analysis and surgical failure

The cumulative probability of surgical success over the 24 month period was evaluated using Kaplan–Meier survival analysis (Figure 1). Surgical success was defined as an IOP between 5 mmHg and 21 mmHg with at least a 20% reduction from baseline and no requirement for secondary glaucoma surgery.

**FIGURE 1:**
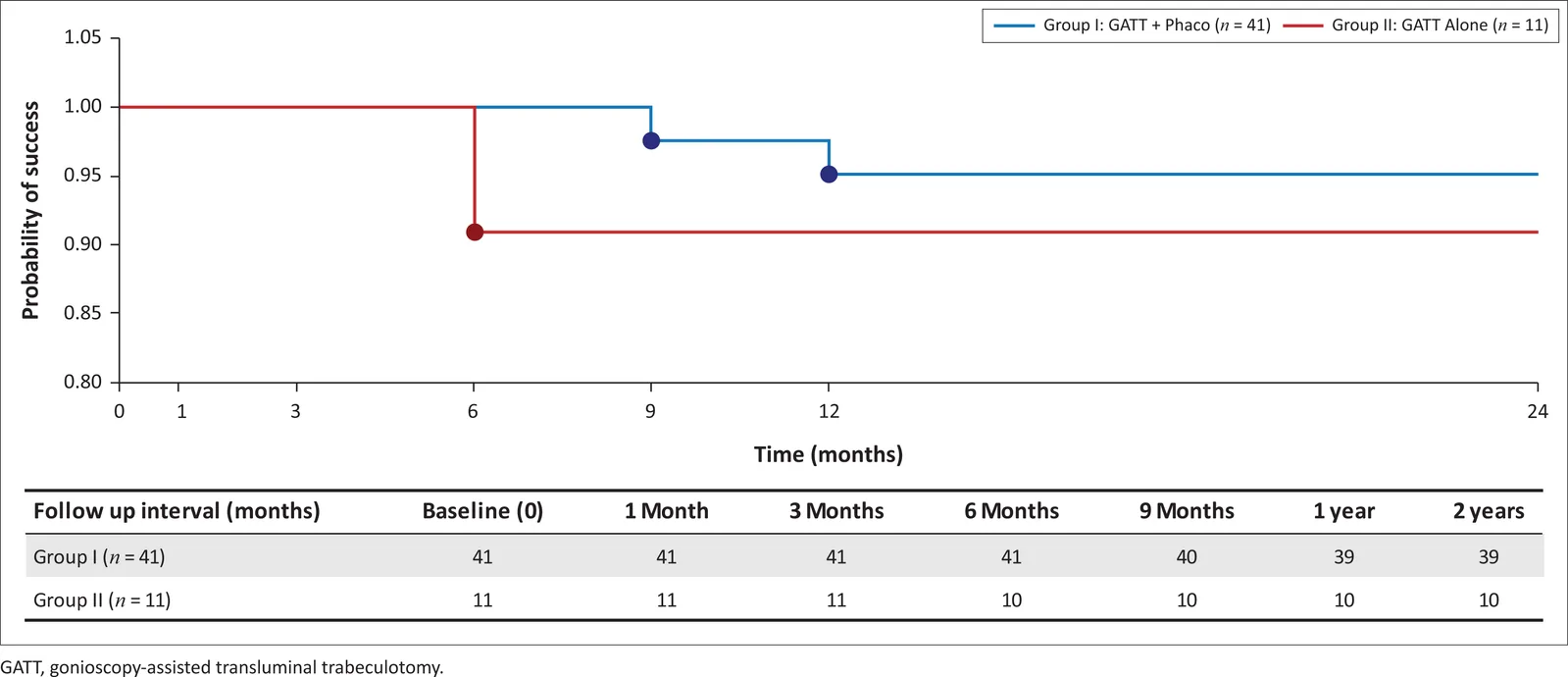
Kaplan–Meier cumulative survival curve.

At the 2-year endpoint, the cumulative survival rate was 95.1% for Group I and 90.9% for Group II. The log-rank test showed no significant difference in cumulative survival between the two treatment cohorts (*p* > 0.05).

Over the 2-year follow-up, three eyes experienced surgical failure due to poorly controlled lOP on maximal medical therapy and required secondary glaucoma interventions. In Group I, two eyes failed: One eye underwent transscleral cyclophotocoagulation (TSCPC) at month 9, and one eye required a trabeculectomy at month 12 (1 year). In Group II, one eye failed at month 6 and was managed with Ahmed glaucoma valve implantation.

## Discussion

The South African patient population is underrepresented in MIGS literature. Given the high prevalence of POAG in South Africa, there is a pressing need for effective, safe, and sustainable surgical options, and studies to determine whether the results align with the outcomes reported in other populations.^5^

In this study, the mean baseline IOP of all 52 eyes was 23.4 mmHg ± 6.4 mmHg, consistent with values reported in earlier GATT series.^25,26,27^ Following surgery, the overall mean IOP decreased from 23.4 mmHg ± 6.4 mmHg preoperatively to 13.8 mmHg ± 4.2 mmHg at 24 months, to 12.1 mmHg ± 3.9 mmHg in the combined group and to 14.2 ± 2.8 mmHg in the standalone group. There was a reduction in the mean number of glaucoma medications from 3.5 ± 0.6 to 2.5 ± 1.1. p. These findings are broadly consistent with those reported by Wan et al., who demonstrated a 2-year mean IOP of 12.6 mmHg ± 2.9 mmHg following combined cataract and GATT procedures, as well as Bektas et al., who reported a 1-year post-operative IOP of 13.8 mmHg ± 4.2 mmHg in standalone GATT alone.^22,28^

The greater IOP reduction observed in the combined group is likely attributable, in part, to the IOP-lowering effect of phacoemulsification, which enhances aqueous outflow by widening the anterior chamber angle and improving trabecular meshwork facility.^15,27^ However, large clinical studies such as the Ocular Hypertension Treatment Study (OHTS) have demonstrated that cataract surgery alone typically produces only modest IOP reductions, averaging 1 mmHg – 2 mmHg, with greater benefit in eyes with higher preoperative pressures.^29,30,31^

Meta-analysis, including that by Friedman et al., report reductions of 2 mmHg – 4 mmHg. This modest effect is far smaller than the reductions observed in our combined group, supporting the interpretation that the enhanced pressure control reflects a true additive effect of trabeculotomy rather than phacoemulsification alone.^32^

Importantly, standalone GATT in our cohort also achieved substantial IOP reduction, supporting its use in phakic patients without visually significant cataracts. These outcomes align with earlier reports by Grover et al. and Rahmatnejad et al., which demonstrated durable pressure-lowering effects in standalone GATT cohorts.^10,27,33^

In addition to effective IOP control, this study documented a significant decrease in medication burden across both groups. In the combined group, the average number of topical medications was reduced from 3.5 ± 0.6 preoperatively to 2.4 ± 1.3 at 24 months, whereas in the standalone group, it declined from 3.6 ± 0.5 to 2.8 ± 0.7. This level of reduction is comparable with the results reported by Guo et al.,^15^ whose meta-analysis revealed consistent reductions in medication dependency following GATT. The reduced need for topical therapy is not only beneficial for patient adherence and ocular surface preservation but also represents a cost-saving advantage, particularly relevant in the South African public healthcare setting.

Regarding safety, the complication profile observed in our cohort aligns well with existing GATT literature, which highlights its minimally invasive nature and generally self-limited complications.^15,25^ Hyphema was the most frequent postoperative finding, occurring in 39.0% of patients in the combined group and 18.2% in the standalone group for microscopic hyphema and macroscopic hyphema in 22.0% and 45.5%, respectively. Importantly, postoperative hyphema is not necessarily detrimental. Grieshaber et al. demonstrated in a canaloplasty cohort that micro-hyphema was associated with improved aqueous outflow and could serve as a positive prognostic indicator of surgical success.^12^ Similarly, Guo et al. in their systematic review and meta-analysis of GATT outcomes reported that transient hyphema was the most common postoperative complication, occurring in approximately 30% – 40% of cases and was almost always self-limiting without long-term sequelae.^15^ Aktas et al. evaluated the episcleral venous fluid wave during GATT and observed that eyes exhibiting postoperative hyphema often had effective trabecular meshwork unroofing and restored aqueous outflow, further supporting the notion that hyphema can indicate procedural success rather than a complication.^25^ In our series, all hyphema cases resolved spontaneously without the need for surgical intervention, and no patients developed persistent hyphema, corneal staining, or high IOP spike, underscoring the generally benign and self-limited nature of this finding.

Other complications, such as fibrinous uveitis, occurred infrequently and responded well to topical corticosteroids. Importantly, no vision-threatening adverse events such as endophthalmitis, persistent hypotony or choroidal detachment were recorded. Moreover, only three patients required further glaucoma surgery during the follow-up period, underscoring the durability and safety of GATT as a primary surgical intervention in POAG.

Additionally, the use of a 5.0 or 6.0 polypropylene suture as the trabeculotome, rather than an illuminated microcatheter, highlights a cost-conscious approach suited for resource-constrained healthcare environments. Despite the lower cost, the suture-based technique achieved comparable IOP and safety outcomes to those reported with microcatheter-assisted GATT, as also described by Bektas et al. and Aktas et al.^28,34^

Over a 2-year follow up, both surgical approaches were associated with significant reductions in IOP and a marked decrease in the need for glaucoma medications. Importantly, the procedure demonstrated a favourable safety profile, with complications that were generally mild, transient, and conservatively managed. These findings contribute to the growing international evidence supporting the role of GATT as a minimally invasive, conjunctiva-sparing alternative to traditional glaucoma surgery.

### Limitations of the study

The findings of this study relate to a cohort of patients who were diagnosed and underwent surgery at a secondary eye care facility in an urban setting in South Africa; therefore, they may not be generalisable to the country as a whole. In addition, the retrospective design, lack of blinding in IOP measurements and relatively small sample size may affect generalisability, particularly the comparison of the subgroups. Prospective, randomised trials are needed to further strengthen the evidence supporting GATT in this population.

## Conclusion

This study demonstrates that GATT, performed either as a standalone procedure or in combination with phacoemulsification, is a safe and effective surgical option for South African patients with POAG. Over a 2-year follow-up period, both approaches achieved substantial and sustained reductions in IOP with low complication rates.

Although IOP outcomes were comparable between the groups, combined GATT and phacoemulsification showed a trend towards lower postoperative medication requirements, highlighting the potential benefit of addressing glaucoma and coexisting cataract in a single surgical session.

These findings support the use of suture-assisted GATT in resource-limited settings and contribute valuable data from South African patients, who remain underrepresented in glaucoma research. Larger prospective studies focusing on South African patients with longer follow-up periods are needed to validate these outcomes and refine surgical strategies for diverse patient populations.
